# Enset Landraces: Conservation, Distribution, and Use in an Enset-Based Agricultural System

**DOI:** 10.1155/sci5/7440580

**Published:** 2025-03-07

**Authors:** Tafesse Kibatu, Tileye Feyissa, Sebsebe Demissew, Diriba Muleta

**Affiliations:** ^1^Department of Horticulture, College of Agriculture and Natural Resources, Dilla University, Dilla, Ethiopia; ^2^Institute of Biotechnology, Addis Ababa University, Addis Ababa, Ethiopia; ^3^Department of Microbial, Cellular and Molecular Biology, College of Natural and Computational Science, Addis Ababa University, Addis Ababa, Ethiopia; ^4^Department of Plant Biology and Biodiversity Management, College of Natural and Computational Science, Addis Ababa University, Addis Ababa, Ethiopia

**Keywords:** enset, enset landraces, *Ensete ventricosum*, false banana, landrace diversity

## Abstract

Enset is a unique food security crop for more than 25% of the population in Ethiopia and serves as a food, animal feed, medicine, and fiber source, with significant resilience and environmental adaptability. Enset growing zones harbor several landraces, resulting from differences in natural selection, human selection, and cultural management practices. Understanding landrace propagation, selection, and in situ conservation practices is crucial. These practices are essential for crop improvement, management, conservation, and enhancement of genetic resources. This study investigates the utilization and in situ conservation practices of enset landraces and draws insights from farmers and agricultural experts through in-depth interviews, surveys, group discussions, and field observations. Representatives from the enset-growing areas of Hadiya, Gedeo, Gurage, and Kembata Tembaro zones and Basketo Special Woreda were selected due to their diverse sociocultural practices, geographic landscapes and distribution, altitude variations, enset farming systems, and rainfall patterns. Purposive sampling was employed to select 19 representatives from woredas based on traditional enset culture practices. Subsequently, 31 kebeles were chosen based on their altitude differences. A total of 406 household units were then selected. The relative density determined the abundance of individual landraces. The distribution and richness of landraces across sites were analyzed using Simpson's diversity, Shannon–Wiener, and Margalef's indices. A total of 274 vernacular names for enset landraces were recorded, but only 106 (39%) were cultivated in the sampled households. Despite this, analysis of diversity indices (Shannon–Wiener, Simpson, and Margalef) revealed no significant differences between the study sites. The analysis also revealed moderate to high enset landrace diversity across the studied sites. The study highlights a rich and diverse collection of landraces adapted to various ecological conditions and fulfilling various purposes (food, feed, fiber, and medicine). Notably, food value emerged as the primary driver of landrace selection and abundance. Promoting sustainable enset agriculture should, therefore, prioritize maintaining landrace diversity and supporting traditional knowledge to enhance resilience and food security.

## 1. Introduction

*Ensete ventricosum* (Welw.), Cheesman, is commonly known as the false banana due to its similarity to the banana plant. It belongs to the family Musaceae. The genus *Ensete* is one of the three genera in the family known for monocarpic flowering, large leaves, and corms. The wild ensets are distributed in tropical regions of Eastern, South, and Central Africa [1, 2]. However, it has been cultivated for thousands of years in the Southern and Southwestern parts of Ethiopia under enset-based agricultural systems. Its cultivation has also partially expanded to Central, South Western, and Western parts of Ethiopia [1, 3–10]. It covers upto 25% of arable lands and is a staple food crop for more than 25% of the population in Ethiopia [11–14].

The main food products of enset are kocho, amicho, and bulla. These products offer a rich nutritional profile, including carbohydrates, minerals, essential amino acids, and dietary fiber [15–17]. Enset is also well known for animal feeds, medicine, and fiber products [18, 19]. Various parts of the enset plant, especially the corm and leaves, are used to treat a wide range of human and animal ailments in traditional medicine, with certain landraces believed to have superior medicinal properties [4, 20, 21]. Its high productivity, ability to be processed at any stage of growth, and long-term storability make enset a unique and staple food crop [22, 23]. Furthermore, it supports larger population per unit area than cereal-based systems due to its high yield per hectare [24].

The enset plant is much more than just a crop in Ethiopia. It is deeply interrelated with the socioeconomic and cultural aspects of the communities, its abundance, a symbol of wealth, and status in some communities [4, 25]. This is reflected in its influences social status through land ownership, features in rituals symbolizing abundance, fertility, and even spiritual connection and plays a role in food culture through the preparation of food [24, 26, 27].

Enset's influence extends beyond food security. It empowers women throughout the production cycle, from planting and harvesting to processing and marketing [28]. According to the study, women even classify enset landraces based on their uses, reflecting the deep local knowledge and cultural significance of this crop. Environmentally, enset farming benefits the soil by reducing erosion, enhancing nutrient cycling, and increasing organic carbon storage [29]. It also mitigates microclimate changes by providing shade and regulating temperature, impacting the performance of other crops [30]. Finally, enset's drought tolerance contributes to food security in areas with low rainfall [29]. Enset has diverse names across languages and features prominently in folktales and proverbs, highlighting its significance in the collective consciousness [4, 25]; Yemata, 2020. These aspects underscore the crucial role of enset in Ethiopian food security and household wellbeing [15, 31].

Greater enset landrace diversity is found in areas where the crop originated and was domesticated. In these areas, the landrace population, associated human knowledge, and social values are all maintained [32, 33]. Understanding the diversity and use of the crop in an area requires understanding the people who grow it, the climate and soil conditions, and the distribution of wild and cultivated landraces [34]. Moreover, the diversity pattern resulted from an interaction between the genetic makeup of the plants, the environmental factors, and human selection and management activities.

Domestication, selection practices, management decisions, and farmer preferences have all influenced enset diversity and genetic resources. The reasons might be due to farmers' multiple criteria, such as food value, disease and pest resistance, and drought tolerance when selecting and ranking landraces [35–37]. The other reason is related to the plant reproduction method and the propagation techniques followed by the farmers. Enset harvesting is performed before flowering and seed set [38, 39]. In addition, slow and poor germination of hard-coated enset leads to vegetative propagation [40]. Thus, the possibility of getting new genetic traits due to seed propagation is very low in the farmers' field.

Enset encompasses a large number of landraces with unique names varying across ethnic groups, sometimes even within administrative zones. The existence of a large number of enset landraces in Ethiopia resulted from differences in their natural selection, human selection, and cultural management practices [39, 41–43]. Botanists use a combination of morphological, genetic, ethnobotanical, and literature-based approaches to accurately identify, classify, and authenticate Ensete ventricosum [1, 4, 44]. Understanding farmers' practices of landrace propagation, selection, and in situ conservation is crucial for crop improvement, management, landrace conservation, and enhancement of genetic resources. The selected study areas, encompassing more than 25% of enset crop growing zones with diverse cultural, ethnic, social, and agroecological groups, have been understudied in previous research [4, 17, 41]. This focus allows for a detailed investigation of landrace distributions and farmer perspectives on landrace utilization. Thus, the objective of this study was to evaluate preferred uses of cultivated enset landraces, analyze in situ abundance and distribution of enset landraces, and assess enset landrace diversity and sources of seedlings.

## 2. Methodology

### 2.1. Description of the Study Sites

**Figure 1:**
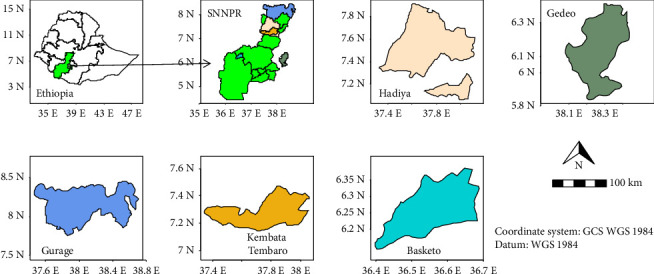
Map of the study sites.

Enset is mainly grown in the Southern, Southwestern, Central, and Western parts of Ethiopia. Five study sites were selected from the southern region: Hadiya, Gedeo, Gurage, and Kembata Tembaro zones and Basketo Special Woreda (Figure 1).

These sites were chosen for their diverse characteristics, including sociocultural practices, geographic landscapes and distribution of enset farming systems, altitude variations, and rainfall patterns. The elevation ranges from 1000 to 3900 m above the sea level, with annual rainfall varying between 600 and 1800 mm. Notably, Gurage zone has the highest and lowest recorded elevations within the study area. Gedeo agriculture is known for its unique UNESCO-recognized complex agroforestry system [45, 46].

Southern Ethiopia is a region brimming with linguistic diversity. Each zone is dominated by a language reflecting the local ethnicity. The Hadiya people speak Hadiyisa, a Cushitic language related to Somali and Oromo. Similarly, Gedeo, another Cushitic language closely tied to Hadiyisa, dominates communication in the Gedeo Zone. The Gurage Zone showcases more diversity belong to the Cushitic branch of the Afroasiatic family. The Kembata Zone presents a different picture. The Kembata people speak Kembataata, an Omotic language distinct from the Cushitic branch. However, in the Alaba and Tembaro areas, Amharic, Ethiopia's national language, holds dominance, although some indigenous languages might persist. The Basketo Special Woreda boasts its own unique language, Basketo [47, 48].

### 2.2. Survey Procedure

**Figure 2:**
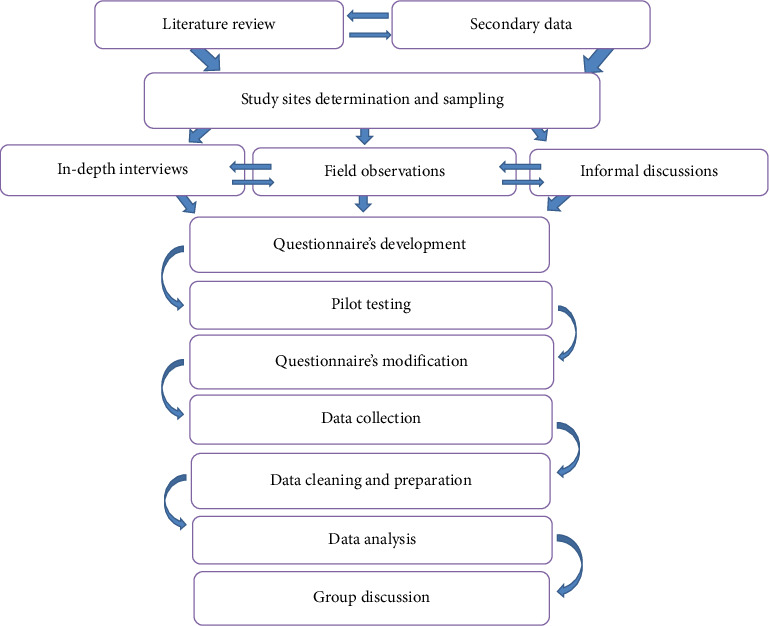
Flowchart of the survey procedure.

The research process began with a comprehensive review of published literature. This initial step provided a strong foundation for understanding enset production and productivity. We then collected secondary data records from zone agriculture offices to assess the potential and status of enset at the potential enset growing areas. Based on this information, we selected study sites, considering factors such as geographic spread, ethical considerations, enset production levels, and landrace diversity. Following the selection of study sites, we proceeded with the sampling process. Then, in-depth interviews were conducted with experienced agricultural experts and farmers. Next, field observations and informal discussions were conducted. Structured and semistructured questionnaires were then developed in Amharic for each study site, ensuring consistency in content while reflecting the unique characteristics of each location. The questionnaires were refined through pilot testing and finalized. With the assistance of agricultural development agents, the survey data were collected, with local language translation provided when necessary. The data were then cleaned, prepared, and analyzed. To further triangulate and clarify the findings, group discussions were conducted with 5-6 participants, including both agricultural experts and farmers (Figure 2; [49–52]).

### 2.3. Sampling Procedure

Purposive sampling was employed to select 19 representatives Woredas based on the traditional enset culture practices. Then, 31 Kebeles (the lowest administrative region) were selected based on their altitude differences (Table 1; [50, 53, 54]).

To represent the three agroecological zones (highland, midland, and lowland), a Kebele from each zone was selected: highland (> 2500 m), midland (1500–2500 m), and lowland (< 1500 m). A total of 406 household units were then selected from these Kebeles (Table 2).

### 2.4. Diversity Metrics

To analyze the extent of on-farm genetic diversity in the enset agriculture system, we employed ecological diversity models. Relative density, Simpson's diversity index, Shannon–Wiener index, and Margalef's index (MI) were used, treating the landraces as distinct species [55–58].

Relative density (RD) was computed using the following equation:

(1) Landraces' RD=Sum of particular landracesSum of all farm presented landraces ×100.

Simpson's diversity index (SDI) was computed using the following equation:

(2) $$\begin{array}{c} \text{SDI}=1-\frac{\sum {\text{⁣}n}_{i}\left(n_{i}-1\right)}{N\left(N-1\right)}, \end{array}$$

where *n*_*i*_ is the total number of individual landraces in each study sites, and N is the total number of the landraces.

The Shannon–Wiener index (H′) was computed using the following equation:

(3) $$\begin{array}{c} \text{The Shannon}-\text{Wiener index }\left(H^{\prime }\right)=-\overset{n}{\underset{i=1}{\sum }}p_{i}*\ln\left(p_{i}\right), \end{array}$$

where *p*_*i*_ = Ni/*N*, Ni is the number of individuals of the landrace *i*, and *N* is the total number of individuals of all landraces in the study sites.

MI was computed using the following equation:

(4) $$\begin{array}{c} \text{MI}=\frac{S-1}{\ln N}, \end{array}$$

where *S* is the number of landraces, and *N* is the total number of the landraces in the study sites.

### 2.5. Statistical Analysis

Descriptive statistics regarding the sociodemographic characteristics of the respondents were summarized. Chi-square analysis was performed on the frequency data to assess the between-site and within-site variations for each diversity index. Cluster analysis was performed to classify enset landraces based on their preferred use at the study sites. Heatmaps and dendrograms were used to identify the common uses of enset landraces across the study sites and to describe their preferred use. Only valid data were considered for analysis. Statistical presentations and analyses were performed using R Version 4.3.1 with the support of various R packages [60].

## 3. Results and Discussion

### 3.1. The Preferred Use of Enset Landraces

**Figure 3:**
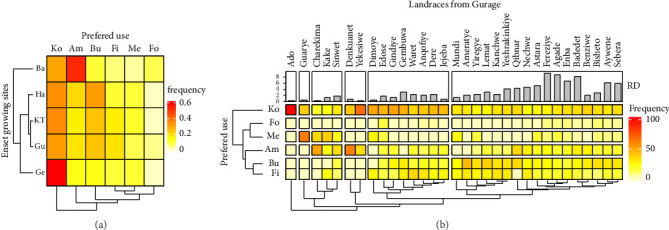
The heat maps illustrate the frequency distribution: (a) preferred use of enset clones at the sampled households and (b) Preferred use of enset at the study sites. The bar plots indicate the evenness of landraces in terms of relative abundance at the study sites. Darker colors represent a higher frequency of a particular landrace being used for a specific purpose. Ko = Kocho; Am = Amicho; Bu = Bulla; Fi = Fiber; Me = Medicinal; Fo = Forage; Ba = Basketo Special Woreda; Ha = Hadiya Zone; KT = Kembata Tembaro Zone; Gu = Gurage Zone; Ge = Gedeo Zone.

Enset crops have diverse uses, serving as food for human consumption, a raw material for industries and construction, animal feed, and even medicinal purposes [28, 60], offering significant socioeconomic and environmental value [26, 29, 61]. Our study in enset-growing regions identified kocho (fermented dough), amicho (boiled corm), bulla (dehydrated juice), fiber, forage, and medicine as the most common uses of enset (Figure 3(a)). Our results align with the reports from Dilebo et al. [62] and Tsehaye and Kebebew [23], highlighting the consistent role of enset across these regions.

Our survey revealed diverse preferences and uses for enset landraces at the study sites. Food emerged as the most highly favored use for these landraces, with kocho being the most preferred food type overall. However, Basketo Special Woreda showed a preference for amicho, while the Gurage Zone favored kocho more. Hadiya Zone displayed a similar preference for both bulla and kocho (Figure 3(a)). Group discussions with agricultural experts and farmers confirmed that landrace selection primarily focuses on food values. This aligns with previous research highlighting food as the predominant value of enset [4, 28, 60, 63, 64]. The prioritization of enset landraces for food over nonfood purposes likely stems from the strong connection between food types and the social, cultural, and economic practices of these communities [62, 63, 65, 66].

For nonfood uses, fiber was the most preferred, followed by medicine and animal feeds. However, in Basketo, feed values were prioritized over the medicinal value. Group discussions with agricultural experts and farmers revealed that farmers primarily select enset landraces based on their food value. There was not a dedicated practice of selecting landraces specifically for nonfood purposes; these benefits were seen as secondary advantages of the landraces chosen for food production. However, some farmers, particularly those known for conserving landraces, valued certain varieties beyond their food value, especially for medicinal purposes (Figure 3(a)). These findings align with other studies reporting fiber as the more emphasized nonfood value, followed by medicinal and feed values [28, 67, 68].

Experts and farmers explained that growing enset for medicinal purposes often requires additional farm activities compared with its less frequent use. This additional workload might discourage farmers from solely cultivating landraces for medicinal purposes. The suitability of a landrace for animal feed depended on the number and types of animals reared, making preference for animal feed directly related to animal husbandry practices. Similarly, the preference for fiber for medicinal and feed uses stemmed from its role as a raw material for various household products and a source of income. Other studies conducted in enset-growing zones have reported similar patterns of nonfood use [39, 68].

Recent applications of enset fiber in medicinal and industrial contexts might increase the commercial and industrial potential of enset landraces beyond their traditional food use. Traditionally, enset fiber has been used to produce ropes, mats, and sacks. It has also been employed locally for construction in roofing and fencing [69]. The versatility of enset fiber has prompted research into various modern applications. As industries seek sustainable alternatives, enset fiber emerges as a renewable resource with low density and favorable mechanical properties, making it a viable alternative to traditional natural fibers [18, 70]. Enset also holds significant potential in modern medicine. Recent studies have begun to explore these traditional uses alongside potential modern applications in the pharmaceutical sector [4, 18, 21, 69]. Recent studies have also begun to explore potential in modern medicine uses alongside potential modern applications in the pharmaceutical sector [4].

Our survey in the Gurage Zone identified 32 enset landraces, categorized into six groups based on their preferred uses in the sampled households (Figure 3(b)). The first group, primarily valued for kocho, includes the Ado landrace. The second group features the Guarye landrace, selected for both its medicinal properties and kocho production. The third group comprises three landraces preferred for kocho, amicho, and medicinal values. The fourth category contains Derkuanent and Yekesiwe landraces, valued for amicho and kocho products. The fifth group consists of landraces appreciated for high kocho value, followed by bulla and fiber. The final group encompasses 17 landraces valued for all three qualities—kocho, bulla, and amicho (Figure 3(b)).

Our findings align with other studies highlighting that most landraces in the Gurage Zone are used primarily for food consumption, with kocho having the highest value, followed by bulla and amicho [62, 71]. Fiber and feed values are reported as the preferred nonfood uses. In the Gurage Zone, 30%–40% of landraces are used for medicinal purposes, with varying levels of farmer acceptance (Figure 3(b); [71]). The cultivation of landraces for medicinal use might be linked to economic status and altitude [62, 71]. The preference for kocho over other uses might be due to its nutritional content, food value, and cultural significance [41, 72].

**Figure 4:**
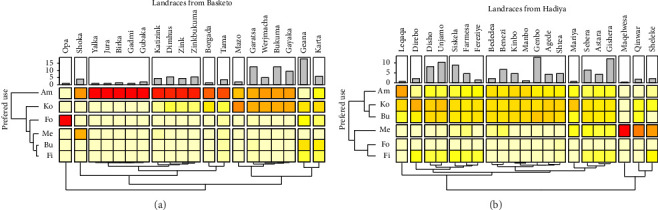
The heat maps illustrate the frequency distribution of preferred use of enset at the study sites. The bar plots indicate the evenness of landraces in terms of relative abundance at the study sites. Darker colors represent a higher frequency of a particular landrace being used for a specific purpose. Ko = Kocho; Am = Amicho; Bu = Bulla; Fi = Fiber; Me = Medicinal; Fo = Forage.

Our survey in Basketo Special Woreda identified 20 enset landraces. Based on their most frequent uses, we categorized them into 10 groups (Figure 4(a)). The first group consists solely of “opa,” a wild enset landrace primarily used as animal fodder. The second group features “amicho” and “Shoka,” both preferred for medicinal purposes. The third group comprises five landraces valued for their “amicho” qualities. The fourth category includes four landraces highly preferred for “amicho,” with additional value for “kocho.” Groups five through eight each contain landraces valued for both “kocho” and “amicho,” with potentially equal importance. The ninth group features “Geana,” a landrace prized for its “kocho,” “bulla,” fiber, and forage value. Finally, the 10th group includes “Karta,” a multifunctional, nonmedicinal landrace (Figure 4(a)).

Our survey in the Hadiya Zone identified 21 landraces categorized into 10 groups based on preferred uses (Figure 4(b)). Laqaqa (Group 1) is valued primarily for kocho but also for amicho, bulla, and fiber. Diriba (Group 2) is valued for kocho, bulla, and fiber. Groups 3 and 4 include landraces preferred for kocho, bulla, amicho, and fiber. Group 4 additionally has three landraces valued for amicho alongside kocho. Group 5 consists of seven food-valued landraces. Mariya (Group 6) is preferred for both food and medicinal purposes. The three landraces in Group 7 have multipurpose uses excluding feed. Maqelwesa (Group 8) is a medicinal landrace. Group 9 comprises medicinal Qinwar landraces additionally valued for kocho and bulla. Finally, Group 10 includes medicinal Sheleke landraces with value for both kocho and fiber. These findings align with reports by Kusse, Ermias, and Darch [73], Yemataw et al. [74, 75], and and Yemata [4].

**Figure 5:**
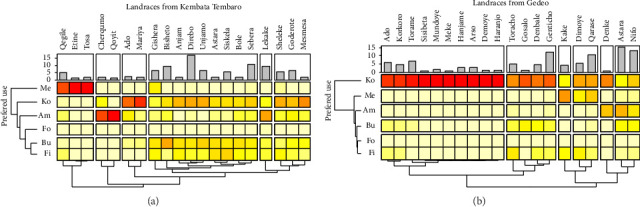
The heat maps illustrate the frequency distribution of preferred use of enset clones at the sampled households. The bar plots indicate evenness of landraces in terms of relative abundance at the study sites. Darker colors represent a higher frequency of a particular landrace being used for a specific purpose. Ko = *Kocho*; Am = *Amicho*; Bu = *Bulla*; Fi = Fiber; Me = Medicinal; Fo = Forage.

Our survey in the Kembata Tembaro Zone identified 20 landraces categorized into six groups based on their use (Figure 5(a)). The first group comprises three landraces valued primarily for medicinal purposes. The second and third groups feature Cherqumo/Qoyi and Ado/Mariya, respectively, both preferred for amicho and kocho. The fourth group contains nine multipurpose landraces. The fifth category includes the Lekake landrace, valued for both food and fiber. The last category comprises three landraces with similar food and fiber value. Consistent with other studies, our findings show enset landraces are primarily used as food. Interestingly, the use of landraces for medicine in Kembata Tembaro Zone was relatively high compared with other zones [68, 71].

Our survey in the Gedeo Zone identified 20 landraces, categorized into six groups based on their use (Figure 5(b)). The first and second groups, comprising ten and four landraces, respectively, are valued primarily for kocho production. Group 3 consists solely of the medicinal landrace Kake. Group 4 features Dimoye and Qarssa landraces, valued for kocho with the added benefits of fiber and medicine. Group 5 includes Derke, valued for both kocho and amicho. Finally, Group 6 encompasses multipurpose Astar and Nifo landraces. Previous studies report that some areas in the Gurage Zone practice enset processing without extracting bulla [68, 71]. Group discussions with participants revealed suggestions that leaving the bulla might improve kocho taste and color.

### 3.2. Enset Diversity and Distribution

#### 3.2.1. Distribution of Enset Landraces

In-depth interviews revealed that participants recognized a total of 259 enset landraces existing at the study sites. A subsequent farm survey identified an additional 15 landraces, bringing the total recorded vernacular names to 274 (Table 3; Supporting Tables 1–5). However, only 106 (39%) of these landraces were actually cultivated in the sampled households. The Basketo Zone maintained the highest proportion (80%) of landraces cultivated from the reported list. The lowest proportion (27%) was found in the Kembata Tembaro Zone, similar to the neighboring Hadiya Zone (30%). Gurage and Gedeo Zones had cultivation proportions of 38% and 53%, respectively (Table 3). These findings align with previous studies by Dilebo et al. [62]; Tsehaye and Kebebew [23]; Yemata [4]; Yemataw et al. [39]; and Zeberga et al. [72].

Landrace vernacular names can reflect places, enset morphology, traditional agricultural practices, and cultural uses [76, 77]. This can lead to the same landrace having different names in different locations and vice versa [28, 75]. Thus, it is ambiguous to determine whether the gap between expected and actual landraces indicates a loss of genetic resources. As a result, the gap between the reported number of landraces and those cultivated does not necessarily indicate a loss of genetic resources. However, group discussions revealed that participants identified nonrandom selection as a possible reason for the gap, supported by findings from Blomme et al. [63]; Borrell et al. [41]; and Yemataw et al. [74]. Therefore, while informal naming practices contribute to the gap, it is also possible that the difference reflects, to some extent, a loss of enset landraces due to preferential selection.

Figure 3(a) shows the abundance (measured by relative density) of individual enset landraces across farms in the Gurage Zone. The highest abundance is found in the fourth cluster, containing Badadet, Agabe, and Fereziye landraces. Conversely, the lowest abundance is observed in the first, third, and sixth clusters, with Ado, Charekima, and Yekesiwe landraces, respectively. Notably, the sixth cluster, with the highest overall abundance, contains landraces valued for food, nonfood, and medicinal purposes. This suggests that farmers' conservation efforts prioritize multipurpose landraces. These findings highlight the importance of considering multipurpose uses in enset breeding and crop improvement programs rather than solely focusing on food value. This aligns with the suggestions from group discussions and comparable studies [62, 63].

Our analysis in Basketo Special Woreda revealed variation in the abundance of individual landraces across sampled household farms (Figure 3(a)). The ninth cluster, containing only the multipurpose Geana landrace, exhibited the highest abundance. Conversely, the lowest abundance was observed in the first and third clusters, which include Opa, Yalkka, Jura, and Gadmi landraces. This pattern suggests a preference for multipurpose landraces, as evidenced by the higher abundance of Geana and landraces in the eighth cluster valued for both amicho and kocho.

The abundance of individual enset landraces (measured by relative density or RD) varied across farms in Hadiya, Kembata Tembaro, and Gedeo Zones (Figures 4(b), 5(a), and 5(b)). In Hadiya Zone, the highest RD was found in the fourth cluster containing Genbo landraces, while the lowest was in the eighth cluster by Maqelwesa (medicinal). Interestingly, the most abundant landraces overall belonged to the third cluster (Disho and Unjamo), followed by the seventh cluster, valued for both food and fiber. A similar pattern emerged in Kembata Tembaro Zone, where the highest RD was observed in the fourth cluster (Direbo landraces), with the lowest in clusters containing Etine and Qoyit landraces. Again, the most abundant landraces were found in clusters with food and fiber value (fourth and fifth). Gedeo Zone exhibited a similar trend, with Astara landraces having the highest RD and Sisibeta and Meki the lowest. Here, the most abundant landraces belonged to the second and sixth clusters. These findings align with previous studies by Blomme et al. [71]; Tsehaye and Kebebew [23]; Yemataw et al. [75, 78]; and Zeberga et al. [72].

As our study and others have shown, a limited number of landraces dominate farms in the sampled households. Our analysis indicates that use value, particularly food value, is a key factor influencing landrace choice and abundance. This aligns with findings from Blomme et al. [71]; Jacobsen et al. [79]; and Shumbulo et al. [80]. However, the diversity of landrace abundance at the individual farm level may also be influenced by socioeconomic conditions, ethnolinguistic background, agroecological conditions, farm size, and biotic and abiotic factors [63, 78, 81].

#### 3.2.2. Geographic Diversity Indices

Analysis of diversity indices in Table 4 reveals moderate to high enset landrace diversity across the five studied sites. This finding aligns with recent studies by [39, 71, 72], who reported relatively high diversity indices. Simpson's index studies at Silte and Hadiya have shown values ranging from 0.963 to 0.978, indicating a high level of diversity across these zones. The Shannon–Wiener indices for these zones ranged from 3.73 to 3.96, indicating high richness and evenness of enset landraces in these areas. This suggests that enset landraces are widespread among farmers, contributing to agricultural resilience and farmers cultivate a wide variety of enset landraces, which can enhance food security and adaptability to changing environmental conditions [17, 37, 72].

Statistical analysis using chi-square tests (*χ*^2^) revealed no significant differences in the Shannon–Wiener diversity index (H′), SDI, and MI between the study sites (Table 4). H′ values ranged from 1.11 to a maximum of 1.15. The Gurage zone had the highest SDI (0.96), while both Hadiya and Gedeo zones had the lowest (0.92). Similarly, the Gurage zone exhibited the greatest landrace richness based on the Margalef index (4.97), while the Kembata Tembaro zone had the lowest (3.08) (Table 4). Collectively, these diversity indices suggest moderate to high landrace diversity and richness across all five sites [82].

### 3.3. Planting Materials for Conservation

Farmers at the study sites propagate enset through vegetative methods from various sources. The primary method (74.10%) involves growing seedlings from existing landraces on their farms. This limits the introduction of new landraces and traits, as enset is propagated asexually and harvested before flowering and seed set [39, 72, 81, 83]. However, alternative sources, such as exchanging planting material with neighbors (16.50%) and purchasing from markets (9.40%), could introduce new genetic traits and contribute to a higher diversity of landraces. Similar practices with varying proportions have been reported in other studies [39, 62, 79, 83]. Participants in the group discussion also emphasized seedling exchange and market purchases as being critical for landrace diversification. While enset can naturally reproduce through seeds, this method is rarely employed by farmers due to harvest timing, limited seed viability, and cultural practices [84].

## 4. Conclusion

Our study documented a significant number of enset landraces, resulting in a high diversity index across the study regions. While the overall diversity was similar, we observed notable variations in landrace distribution and uses between regions. Food value was the primary driver for landrace selection and abundance, with kocho being the most preferred food product. Fiber was the most sought-after nonfood use due to its versatility and income generation potential. However, the limited exchange of planting materials hindered the introduction of new genetic resources and the increase of landrace diversity at individual farms. Understanding the diverse uses, selection criteria, and sources of enset landraces is crucial for developing sustainable and climate-resilient agricultural practices. Future research should prioritize investigating the genetic diversity of these landraces using molecular markers to identify unique genetic resources. In addition, a comparative analysis of the nutritional composition of different landraces could provide valuable insights for breeding programs. By understanding the ecological factors influencing landrace distribution and adaptation, we can develop climate-resilient varieties that meet the needs of local communities.

## 5. Future Scope

This study offers valuable insights into enset crop conservation, distribution, and usage patterns. However, limitations inherent to surveys, such as potential bias and challenges with interpreting local names, restrict our ability to draw definitive conclusions about these patterns. To gain a clearer understanding of enset landrace distribution, future research employing detailed field observations is recommended. This could involve in-depth examination of enset landraces on participating households' farms, along with a deeper exploration of local nomenclature, considering both the linguistic and social aspects of naming practices. In addition, developing a clear classification system for enset landraces based on local names and their social significance would be beneficial for further biological studies.

Furthermore, to achieve a deeper understanding of the enset-based agricultural system and its relationship to landrace conservation, future research could explore the genetic bases for landrace diversity and investigate the socioeconomic factors influencing landrace preferences. Our study also identified a potential gap between farmer priorities for landrace selection and long-term conservation goals. To address this, we recommend the involvement of public-funded institutions and NGOs in complementing farmer-driven in situ conservation efforts. These organizations can play a crucial role by prioritizing the conservation of landraces that might not be a top priority for farmers based solely on economic and cultural considerations. This collaborative approach ensures the preservation of the entire enset ventricosum gene pool for future generations.

## Figures and Tables

**Table 1:** Study sites samples coverage by Kebeles.

Study sites	Agroecologic zone	Representative Woredas	Representative Kebeles	Sampled households	Participated professionals
Basketo special woreda	High-lands	Basketo	Sasamalesa	34	9
	Low-lands	Basketo	Aobicha, Garawelayita	25	
	Mid-lands	Basketo	Gezeayima	21	

Gedeo zones	High-lands	Bule	Sika	23	8
	Low-lands	Dilla Zuria	Chichu, Gosa	25	
	Mid-lands	Bule	Daro	26	

Gurage zones	High-lands	Gumer, Sodo	Arektshleko, Arma, Dame, Gereno Betet Wenz, Isenna Adangazo, Zizenchona Teredo	30	7
	Low-lands	Abeshege	Jejebena Gasore	29	
	Mid-lands	Cheha, Muhur NA Aklil	Ginab, Yeferezye	30	

Hadiya zones	High-lands	Misha	Shiro	31	8
	Low-lands	Gombora	Lambuda	23	
	Mid-lands	Lemmo	Ashe Kubega	25	

Kembata tembaro zones	High-lands	Kedida Gamela	Taza Gerba	24	9
	Low-lands	Kacha Bira	Gemesha	23	
	Mid-lands	Kedida Gamela	Abomsa, Jore	37	

**Table 2:** Characteristics of the study participants.

Household characteristics	Category	Number of samples (percentages)				
		Basketo	Gedeo	Gurage	Hadiya	Kembata Tembaro
Gender	F	27 (33.8)	23 (31.1)	2 (2.2)	18 (22.8)	26 (31)
	M	53 (66.2)	51 (68.9)	87 (97.8)	61 (77.2)	58 (69.0)

Land size (ha)	≤ 0.5	27 (33.8)	17 (23.0)	42 (51.9)	66 (86.8)	18 (21.4)
	(0.5, 1]	20 (25.0)	17 (23.0)	23 (28.4)	8 (10.5)	40 (47.6)
	(1, 1.5]	23 (28.7)	23 (31.1)	11 (13.6)	2 (2.6)	15 (17.9)
	> 1.5	10 (12.5)	17 (23.0)	5 (6.2)	0 (0.0)	11 (13.1)

Years of formal education	≤ 5	59 (73.8)	49 (66.2)	50 (56.2)	47 (59.5)	55 (65.5)
	(5, 10]	18 (22.5)	21 (28.4)	33 (37.1)	32 (40.5)	15 (17.9)
	> 10	3 (3.8)	4 (5.4)	6 (6.7)	0 (0.0)	14 (16.7)

Family size	≤ 5	19 (23.8)	0 (0.0)	53 (59.6)	15 (19.0)	10 (11.9)
	(5–10]	59 (73.8)	68 (91.9)	36 (40.4)	59 (74.7)	55 (65.5)
	> 10	2 (2.5)	6 (8.1)	0 (0.0)	5 (6.3)	19 (22.6)

Age	≤ 35	18 (22.5)	6 (8.1)	8 (9.0)	6 (7.6)	9 (10.7)
	(35, 50]	50 (62.5)	56 (75.7)	48 (53.9)	58 (73.4)	29 (34.5)
	(50, 65]	9 (11.2)	12 (16.2)	25 (28.1)	15 (19.0)	38 (45.2)
	> 65	3 (3.8)	0 (0.0)	8 (9.0)	0 (0.0)	8 (9.5)

**Table 3:** Number of recognized landraces of enset crop at the study sites.

The study sites	Number of enset landraces				
	Mentioned but unavailable	Not mentioned but available	Mentioned and available	Available at the farms	Known to exist
Gurage zone	48	4	25	29	77
Gedeo zone	17	2	17	19	36
Kembata tembaro zone	58	3	18	21	79
Hadiya zone	40	6	11	17	57
Basketo special Woreda	5	0	20	20	25
Total	168	15	91	106	274

**Table 4:** Comparison of Simpson's, Shannon–Wiener's, and Margalef's diversity indices among the study areas.

Diversity indices	Enset growing sites					Chi-squared (*χ*^2^)
	Hadiya	Kembata	Gurage	Gedeo	Basketo	
Shannon	2.75	3.08	3.86	2.99	3.59	4.00^ns^
Simpson	0.92	0.93	0.96	0.92	0.93	0.99^ns^
Margalef	3.50	3.08	4.97	3.09	2.97	4.00^ns^

*Note:* ns indicates nonsignificant difference.

## Data Availability

The raw data and additional information could be made available from the corresponding author upon reasonable request.

## Supporting Information

Supporting information provides detailed information on the distribution of enset landraces across several zones and a special Woreda in Ethiopia with the survey questionnaire. These tables offer a comprehensive overview of the diversity of enset landraces within these regions, complementing the main manuscript's findings. Specifically, the supporting information includes five tables detailing the list and distribution of enset landraces.
