# Supplementary material for: Enset Landraces: Conservation, Distribution, and Use in an Enset-Based Agricultural System
Source: Scientifica (Cairo). 2025 Mar 7;2025:7440580. doi: 10.1155/sci5/7440580 (PMC11986960; doi:10.1155/sci5/7440580)
Supplement: Supporting Information — Additional supporting information can be found online in the Supporting Information section. [file 7440580.f1.docx]

# Supplementary tables and Questionnaire

Supplementary table 1: List and distribution of enset landraces’ in Gedeo Zone

| **Mentioned to exist**  **but unavailable on sampled farms** | **Not Mentioned**  **but available on sampled farms** | **Mentioned to exist**  **and available on sampled farms** |
| --- | --- | --- |
| Burtukan, Chache, Dine, Fokone, Gecha, Genta, Gulume, Hagena, Harame, Koshe, Medelacho, Mekore, Shana, Tilila, Tilo, Wekeso, Welale (n = 17) | Arso, Hanjame ( n = 2) | Ado,Astara,Demoye,Denbale,Denke,Dimoye,Genticho,Gosalo,Haranjo,Kake,Korkoro,Meke,Mundoye,Nifo,Qarase,Sisibeta,Toracho,Torame (n = 18) |

Supplementary table 2: List and distribution of enset landraces’ in Basketo Special Woreda

| **Mentioned to exist**  **but unavailable on sampled farms** | **Not Mentioned**  **but available on sampled farms** | **Mentioned to exist**  **and available on sampled farms** |
| --- | --- | --- |
| Dasiqanda, Kake, Mosi, Weyshita (n = 4) | None Found | Birka, Borgada, Bukuma, Dimhus, Gadmi, Garatsa, Gayaka, Geana, Gubaka, Jura, Karta, Katizink, Mazo, Opa, Shoka, Tama, Werjmacha, Yalka, Zink, Zinkbukuma (n = 20) |

Supplementary table 3: List and distribution of enset landraces in Gurage Zone

| **Mentioned to exist**  **but unavailable on sampled farms** | **Not Mentioned**  **but available on sampled farms** | **Mentioned to exist**  **and available on sampled farms** |
| --- | --- | --- |
| Achor, Adisamba, Agurkanchwe, Akefoyet, Anzila, Ashinagu, Atiketiye, Bazeryet, Beresiye, Bisha, Bishoamehu, Buaseret, Buhute, Chamiwe, Dedeaso, Demiyet, Demorij, Einiyet, Gembeziye, Geziwet, Ginchewe, Gogot, Guadameratye, GuadKanchwe, Guadra, GuadyeshraKinkeye, Kekoriya, Konchiro, Kuashikuashiye, Mesmesa, Oot, Oret, Siguaner, Sinwet, Teteret, Tobore, Toriye, Welanche, Wenadiye, Yedibiriye, Yefekir, Yeqechrye, Yeshrafireye, Zigeziwe, Zobir ( n = 45) | Dimoye, Edose, Kake, Mundi ( n = 4) | Ado, Agade, Ameratye, Anqofiye, Astara, Aywene, Badedet, Benziwe, Bisheto, Charekima, Denkuanet, Dere, Enba,Fereziye,Gembuwa,Gindiye,Guarye,Jejeba,Kanchwe,Lemat,Nechwe,Qibnar,Sebera,Sinwet,Waret,Yekesiwe,Yeshrakinkiye,Yiregye (n = 28) |

Supplementary table 4: List and distribution of enset landraces’ in Kembata Tembaro Zone

| **Mentioned to exist**  **but unavailable on sampled farms** | **Not Mentioned**  **but available on sampled farms** | **Mentioned to exist**  **and available on sampled farms** |
| --- | --- | --- |
| Abetmarza, Agane, Argema, Ashura, Azugerezu, Bededeana, Belekicho, Bifabelo, Bunache, Dagicho, Dego, Degomarza, Derga, Deriketa, Desho, Edo, Gagabe, Ganticho, Genbo, Genersa, Gina, Ginejere, Guhimso, Gunjena, Gute, Halegena, Hanamuje, Henuwa, Kembaticho, Kesete, Ketketa, Lokenda, Manduluka, Mati, Menara, Moche, Najewera, Oniya, Orike, Segile, Serare, Serpe, Sestebo, Sheret, Tobore, Torore, Wachso, Wahe, Wankebere, Wankorote, Watro, Welanche, Welegele, Weniya, Werkewa, Weshmede, Weshmena, Zebiro ( n = 58) | Ado, Bole ( n = 2) | Anjam,Astara,Bisheto,Cherqumo,Direbo,Etine,Gishera,Goderote,Lekake,Mariya,Mesmesa,Qegile,Qoyit,Sebera,Sheleke,Siskela,Tosa,Unjamo ( n = 18) |

Supplementary table 5: List and distribution of enset landraces’ in Hadiya Zone

| **Mentioned to exist**  **but unavailable on sampled farms** | **Not Mentioned**  **but available on sampled farms** | **Mentioned to exist**  **and available on sampled farms** |
| --- | --- | --- |
| Alebite, Aniya, Awonda, Azguruz, Begeja, Benade, Beneja, Birwesa, Bosora, Buchefere, Dantito, Etesa, Gekena, Geriya, Gomrasa, Hanzena, Haywona, Hinza, Kekera, Keseta, Korete, Lechubo, Luwandiya, Manduluka, Mekehilwesa, Merza, Michorera, Minadee, Moche, Ososa, Oyina, Qogeho, Qombotra, Quina, Senbeto, Shitadana, Soqoo, Tegedad, Torora, Zobira ( n = 40) | Benezi, Farmesa, Fereziye, Kinbo, Qinwar, Shtea (n = 6) | Agede,Astara,Bededea,Direbo,Disho,Genbo,Gishera,Leqaqa,Manbo,Maqelwesa,Mariya,Sebera,Sheleke,Siskela,Unjamo (n = 15) |

**ለእንሰት አምራቾች የተዘጋጀ የምርምር መጠይቅ**

**(Questionnaire for Enset Producers)**

ቀን**፡---------------------**

**(Date)**

ይህ መጠይቅ በእንስት ጋር በተያያዝ ለሚሰራ የዶክትሬት ትምህርት ምርምር የሚውል ነው። የምንሰበስበው መረጃ ከእንሰት ዝርያ ጥበቃ፣ አጠቃቀም እና ስርጭት ጋር በተያያዘ የሚቀርብ ነው። እናንተ ከልምድ የምትሰጡን መረጃ ለምርምሩ ከፍተኛ እስተዋጾ ያደርጋል። የግል መረጃቹ ለማንም አካል ተላልፎ እንደማይሰጥ እና መረጃውንም ለትምህርት ጉዳይ ብቻ እንደምንጠቀመው እናረጋግጣለን። ጊዜያቹን ሰውታቹ ለሰጣቹን መረጃ እናምሰግናለን።

This interview is part of a PhD research project focused on enset plant. We're collecting primary data on enset landrace conservation, distribution, and usage practices. Your insights and experiences will be invaluable to our research. Please be assured that your identity and personal information will remain strictly confidential. Thank you for your time and cooperation.

1. ዞን/ስፔሻል ወረዳ፡________________________

**(Zone/ Special Woreda)**

1. ቀበሌ፡________

**(Kebele)**

1. እድሜ፡_____

**(Age)**

1. የመሬት መጠን/ስፋት፡________

**(Land size)**

1. ጾታ፡__________

**(Sex)**

1. የቤተሰብ ብዛት፡__________

**(Number of family)**

1. የትምህርት ደረጃ፡___________

**(Education level)**

1. የግብርና ዘዴ ዓይነት:-

**(Types of agricultural production system)**

ሀ) እንሰት መሰረት ያደረገ ሐ) አርብቶ አደር

(Enset based) (Animal production based)

ለ) የአዝርት ሰብል መ) ከፊል አርብቶ አደር

**(Cereal based) (Production system)**

ሠ) ሌላ ዓይነት ካለ ይጠቀስ፡___________________________________

**Other (please specify)**

1. እንስት የምግብነት ጥቅሙ:

(Mention and describe any special nonfood use of enset)

__________________________________________________________________________________________________________________________________________________________________________________________________________

1. እንስት ከምግብነት ውጭ ያለው ጥቅሙ:

(Mention and describe any special nonfood use of enset)

____________________________________________________________________________________________________________________________________________________________________________________________________________

1. በማሳ የሚተከል የእንሰት ችግኝ ምንጭ ምንድን ነው?

(Sources of seedlings)

ሀ/ ከማሳ ለ/ ከጫካ እንሰት ሐ/ ከሌላ ቦታ በመግዛት

(Own field) (From wild) (Purchasing)

መ/ ከግብርና ቢሮ ሠ/ መንግስታዊ ካልሆኑ ድርጅቶች

(Agriculture office) (NGOs)

ረ/ ሌላ ምንጭ ካለ ይጠቀስ ____________________________________________

(Other (please specify)

1. የአየር ንብረት

(Agroecology)

ሀ/ ቆላ ለ/ ወይና ደጋ

(Lowlands) (Midlands)

ሐ/ ደጋ

(Highlands)

| በማሳ ላይ የሚገኝ የእንሰት ዝርያ ስም  (Name of an existing landrace) | ተመራጭ ጥቅም (ለቡላ፣ ለቆጮ፤ አሚቾ፣ መድሃኒት፤ ለቃጫ )  Preferred use (Bulla, Kocho, Amicho, medicinal, animal feed) |
| --- | --- |
|  |  |
|  |  |
|  |  |
|  |  |
|  |  |
|  |  |
|  |  |
|  |  |
|  |  |
|  |  |
|  |  |
|  |  |
|  |  |
|  |  |
|  |  |
|  |  |
|  |  |
|  |  |
|  |  |
|  |  |
|  |  |
